# Hypercalcemia, Renal Failure, and Skull Lytic Lesions: Follicular Lymphoma Masquerading as Multiple Myeloma

**DOI:** 10.1177/2324709613486356

**Published:** 2013-04-12

**Authors:** Muhamad Alhaj Moustafa, Justin L. Seningen, Hayan Jouni

**Affiliations:** 1 Division of Hematology and Oncology, Department of Internal Medicine, Mayo Clinic, Rochester, MN; 2 Department of Laboratory Medicine and Pathology, Mayo Clinic, Rochester, MN; 3 Department of Internal Medicine, Mayo Clinic, Rochester, MN

**Keywords:** bone lytic lesions, follicular cell lymphoma, hypercalcemia, multiple myeloma, renal failure

## Abstract

The findings of hypercalcemia, skull lytic lesions, and renal failure are usually characteristic for multiple myeloma. We herein describe an interesting case of B-cell follicular lymphoma that presented with many features mimicking multiple myeloma.

## Case Description

A 69-year-old woman with a past history of coronary artery disease, hypertension, and dyslipidemia was admitted for rapidly progressive muscle weakness. She was previously asymptomatic until 8 weeks prior to admission when she started having predominantly proximal muscle weakness, mild back pain, poor appetite, and dysgeusia. She reported losing 30 to 40 pounds over the same time period. On admission, physical examination showed marked hepatosplenomegaly. Laboratory studies were significant for a severely elevated serum total calcium of 16.8 mg/dL and creatinine of 3.5 mg/dL indicative of acute renal injury. Her hemoglobin and platelets were mildly reduced at 10.4 g/dL and 117 × 10^9^/L, respectively. Subsequent testing revealed suppressed parathyroid hormone (PTH) levels, negative PTH-related peptide (PTHrP), mildly elevated lactate dehydrogenase, and almost 5 times the upper range of normal for serum angiotensin-converting enzyme (ACE) activity along with elevated levels of vitamin D metabolites.

**Figure 1.**
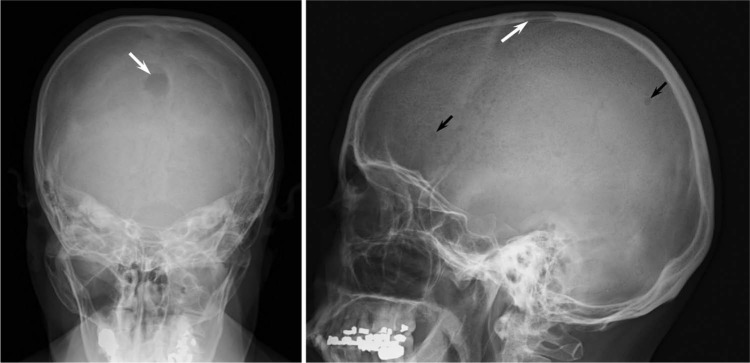
Skull radiographs. A well-defined 1.7-cm lytic lesion in the vertex of the skull was noted (white arrow) in addition to several other smaller lytic lesions (black arrows) in the calvarium suggestive for possible myelomatous involvement.

**Figure 2.**
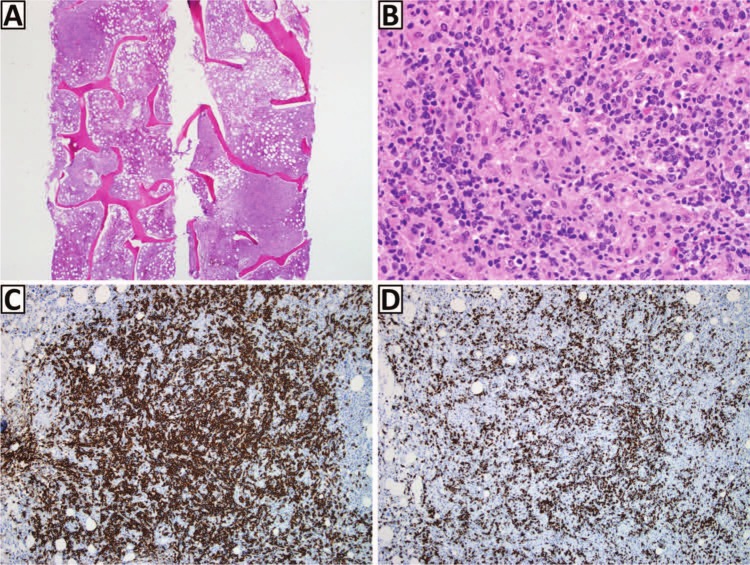
Bone marrow biopsy. A nodular lymphoid infiltrate occupies the marrow interstitium, seen on low magnification, top left and bottom right (A; hematoxylin–eosin [H&E], 20×). High magnification of a nodule reveals intermediate-sized atypical lymphocytes with prominent nucleoli admixed with epithelioid histiocytes with eosinophilic cytoplasm, eosinophils, and small lymphocytes (B; H&E, 400×). Immunohistochemistry demonstrates expression of CD20 (C; CD20, 100×) and BCL2 (D; BCL2, 100×) by the atypical lymphocytes. These findings support the diagnosis of B-cell lymphoma with a prominent histiocytic reaction.

**Figure 3.**
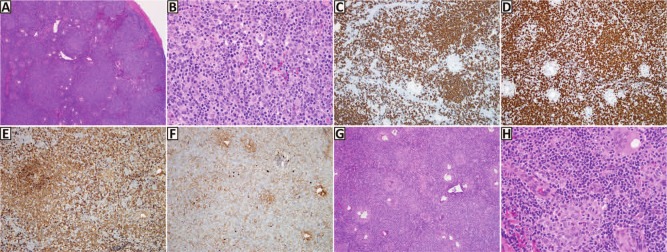
Right inguinal lymph node biopsy. Low magnification shows effacement of lymph node architecture by a vaguely nodular lymphoid infiltrate (A; hematoxylin–eosin [H&E], 40×), which on high magnification is composed of intermediate-sized atypical lymphocytes with prominent nucleoli (B; H&E, 400×). Immunochemistry demonstrates that the tumor cells are positive for CD20 (C; CD20, 100×) and BCL2 (D; BCL2, 100×); they also show Kappa light chain restriction (E, Kappa, 100×; F, Lambda, 100×). These findings are diagnostic of follicular lymphoma. In the interfollicular areas, the lymph node also demonstrates admixed histiocytes, foreign-body type multinucleated giant cells, and granuloma formation, seen at lower magnification (G; H&E, 100×). Higher magnification shows granuloma formation by epithelioid histiocytes (bottom center) and a foreign-body-type multinucleated giant cell (top right) (H; H&E, 400×).

Given strong suspicion for multiple myeloma, she underwent a skeletal survey, which revealed a 1.7-cm punched-out lytic lesion of the vertex of the skull (Figure 1). Interestingly, both serum and urine protein electrophoresis with immunofluorescence failed to demonstrate a monoclonal spike or clonal light chains. Serum free light chains demonstrated an elevated serum Kappa free light chain concentration at 32.1 mg/dL (reference range = 0.33-1.94 mg/dL) and a normal Lambda free light chain concentration at 1.35 mg/dL (reference range = 0.57-2.63 mg/dL) along with an imbalanced Kappa/Lambda free light chain ratio of 23.8. A bone marrow biopsy with flow cytometry studies were performed and surprisingly showed a low-grade B-cell lymphoma with a prominent histiocytic reaction (Figure 2). Subsequently, she underwent a lymph node biopsy, which revealed grade 3B follicular lymphoma (Figure 3). Further staging was performed with positron emission tomography, which showed diffuse patchy uptake within the proximal appendicular and axial skeleton in addition to increased uptake of the large lytic lesion of the skull. The patient was treated with aggressive hydration and Zoledronate with resolution of her hypercalcemia and acute renal injury. R-CHOP chemotherapy was later initiated with significant improvement of her other presenting symptoms.

## Discussion

Hypercalcemia in the setting of B-cell lymphoma can result from various mechanisms. Malignancy-associated hypercalcemia is usually categorized into calcitriol-mediated hypercalcemia, PTHrP-induced hypercalcemia, and local osteolytic hypercalcemia.^1,2^ In our patient, it seems that the main cause of hypercalcemia was mainly increased calcitriol production by the malignant lymphoma cells. However, the increased ACE serum activity and the finding of granuloma formation by epithelioid histiocytes may suggest that these activated lymphoma-neighboring histiocytes may also be contributing to our patient’s hypercalcemia.

B-cell lymphomas rarely present with hypercalcemia, renal failure, and lytic bone lesions, which are usually characteristic for multiple myeloma.^3^ Negative serum and urine protein electrophoresis in the setting of hypercalcemia, lytic bone lesions, and renal failure should always prompt physicians to consider alternative diagnoses such as lymphoma. Differentiation between these 2 conditions cannot be made on the basis of imaging or general laboratory studies. Bone marrow biopsy and lymph node sampling is always necessary to make the final diagnosis.
